# Association between halitosis and female fecundability in China: a prospective cohort study

**DOI:** 10.1186/s12884-021-04315-1

**Published:** 2021-12-20

**Authors:** Xiaona Huo, Lin Zhang, Rong Huang, Jiangfeng Ye, Yulin Yang, Hao Zhang, Jun Zhang

**Affiliations:** 1 Obstetrics and Gynecology Department, International Peace Maternity and Child Health Hospital of China, Shanghai Jiao Tong University School of Medicine, Shanghai, 200030 China; 2 MOE-Shanghai Key Laboratory of Children’s Environmental Health, Xinhua Hospital, Shanghai Jiao Tong University School of Medicine, 1665 Kong Jiang Road, Shanghai, 200092 China; 3 Obstetrics Department, International Peace Maternity and Child Health Hospital of China, Shanghai Jiao Tong University School of Medicine, Shanghai, 200030 China; 4 Department of Maternal and Child Health Care, Shanghai Municipal Health and Family Planning Commission, 300 Expo Village Road, Shanghai, 200125 China; 5 Department of Preventive Dentistry, Shanghai Stomatological Hospital, Fudan University, 356 East Beijing Road, Shanghai, 200001 China; 6 Hainan Women and Children’s Medical Center, Haiko, Hainan China

**Keywords:** Halitosis, Oral hygiene, Oral health, Fecundability, Time to pregnancy

## Abstract

**Background:**

Periodontal diseases and poor oral hygiene are potentially associated with decreased female fecundability. Fecundability refers to the probability of conception during a given period measured in months or menstrual cycles. This study aims to examine whether halitosis is associated with female fecundability in a large sample of Chinese women who planned to be pregnant.

**Methods:**

In 2012, a total of 6319 couples came for preconception care in eight districts in Shanghai, China and were followed by telephone contact. Three thousand nine hundred fifteen women who continued trying to be pregnant for up to 24 months remained for final statistical analyses. Halitosis was self-reported at the preconception care visit. Time to pregnancy (TTP) was reported in months and was censored at 24 months. Fecundability ratio (FR) was defined as the ratio of probability of conception among those with and without halitosis. FR and 95% confidence interval (CI) were estimated using the discrete-time Cox model.

**Results:**

80.1 and 86.1% of women had self-reported clinically confirmed pregnancy within 12 and 24 months, respectively. Halitosis was reported in 8.7% of the women. After controlling for potential confounders, halitosis was associated with a reduced probability of spontaneous conception (for an observation period of 12 months: adjusted FR 0.82, 95% CI 0.72–0.94; for an observation period of 24 months: adjusted FR 0.84, 95% CI 0.74–0.96).

**Conclusions:**

Halitosis is associated with reduced fecundability in Chinese women.

**Supplementary Information:**

The online version contains supplementary material available at 10.1186/s12884-021-04315-1.

## Background

Time to pregnancy (TTP) defined as the time from removing the contraceptive measures to achieving conception with timely intercou**r**se, has been widely used as an informative measure of couple fertility in epidemiological studies [1]. A TTP longer than 12 months is a usual cut-off to define infertility [2] though a 24-month cut-off of TTP for infertility is also used [3]. Infertility is defined as couples having had unprotected sex but failed to conceive naturally for 12 or 24 months [2, 3]. Extensive evidence has shown that advanced woman’s age, smoking, drinking, obesity, endocrine disorders and environmental factors are associated with prolonged TTP and infertility [4–8].

Recently, three epidemiologic studies suggested that periodontal diseases (PD) and poor oral hygiene were also associated with increased TTP [9–11]. It was postulated that bad oral health may exert adverse endometrial effects through systemic inflammation [9]. However, little is known about the association between oral health problems and TTP in Asian populations.

Halitosis, commonly called “bad breath” or “oral malodor”, is defined as an unpleasant or offensive odor mostly arising from the oral cavity [12, 13]. It is prevalent worldwide and affects all ages [14, 15]. More than 80% of the cases had intra-oral causes a, such as poor oral hygiene, gingivitis, periodontitis and dry mouth [13, 16–18]. Keeping clean and healthy oral conditions, as well as periodontal treatment have been shown to be the most basic and effective methods to prevent and reduce halitosis [19]. Therefore, we hypothesize that halitosis might be a proxy for overall poor oral health. This study aimed to investigate the association between halitosis and female fecundability in a prospective study in Chinese women.

## Methods

### Study design and study population

In recent years, the Chinese government has been promoting preconception care for couples who plan to be pregnant. In the Shanghai municipality, China, each district has a designated preconception care clinic usually located in a maternity care facility. Registered residents in the corresponding district may obtain a voucher from their community to receive free care in the designated clinic. At the visit, couples are first informed by health care workers on the purpose and procedures involved in the preconception care and are asked to sign a consent form. Information on demographic characteristics, medical history, family history, health behavior and lifestyle are collected. Physicians conduct a physical examination on the couples. Routine blood and urine tests are performed. Couples are provided with health education on how to prepare a healthy pregnancy such as folate supplementation and smoking cessation. After the blood and urine test results are obtained, physicians provide a risk assessment and counseling to the couple. Women are contacted by health care workers via telephone calls at 3, 6, 12 and 24 months after the care encounter. If a woman becomes pregnant, information on pregnancy and pregnancy outcomes is collected. Women were asked “Have you had a clinically diagnosed pregnancy? For how many months have you tried to conceive?”

Eight out of 17 Shanghai districts were randomly selected in an evaluation study by the municipal government. The purpose of the study was to evaluate the benefits and effectiveness of the free preconception care program. The current analysis utilized the data from the evaluation study.

**Fig. 1:**
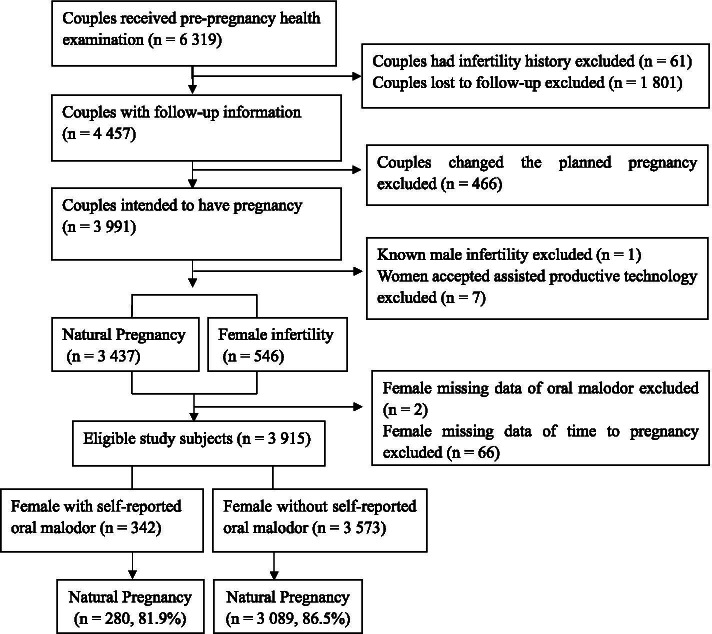
Study Flow Chart

From January 2012 to December 2012, a total of 6319 couples sought preconception care at these clinics. Exclusion criteria were couples who 1) had infertility history; 2) were lost to follow-up; 3) changed the pregnancy plan; 4) were diagnosed with male infertility; 5) accepted assisted reproductive technology; 6) had missing data on oral malodor; 7) had missing data on TTP. Figure 1 illustrates the sample selection process. In the end, 3915 couples remained for final analyses. Among them, 3369 (86.1%) women had given birth or were pregnant at the time of the final follow-up at 24 months while 546 (13.9%) women had not been pregnant during the entire follow-up period. This study was approved by the Ethics Committee of Xinhua Hospital Affiliated to Shanghai Jiao Tong University School of Medicine, Shanghai, China.

### Measures

Halitosis was a self-reported item in the questionnaire with responses of yes/no. Halitosis refers to unpleasant odor currently originating from mouth that was detected by herself or others, or diagnosed clinically, and regardless of whether it was acute or chronic. The outcome was natural conception within the status of pregnancy at 12 and 24 months. TTP was defined as the number of months that a couple were trying to conceive spontaneously.

### Statistical analysis

Bivariate analyses were conducted to assess the association between socio-demographic characteristics and halitosis by Chi-square tests and Mann-Whitney U tests. Therefore, fecundability ratio (FR) and 95% confidence interval (CI) for halitosis were calculated in the Cox proportional hazard model for discrete-time survival analysis. A FR < 1 represents reduction in fecundability, and a FR > 1 represents higher fecundability. The TTP was censored at 12 or 24 months in the corresponding analyses, respectively.

A set of potential confounders were selected based on a directed acyclic graph (DAG, Supplementary Fig. 1) in consideration of previous reports [12], including female age at preconception care (< 25, 25–30, 30–35, 35–40, ≥ 40 years), BMI at preconception care (< 18.5, 18.5–24.9, 25–29.9, ≥ 30 kg/m^2^), indicators of socioeconomic status (female occupation [teachers, civil servants and businessmen; farmers, workers and waiter; housewife or others] and education level [≤ 9, 10–12, 13–16, ≥ 17 years]), smoking status (yes, no, unknown), alcohol consumption (yes, no, unknown), diet (dislike to eat vegetables and like to eat raw meat), indicators of psychological stress (perceived life or work-related stress; tension with relatives, friends or colleagues; and financial pressure), medical history (including anemia, hypertension, heart disease, diabetes, thyroid disease, chronic nephritis, tumor, tuberculosis and hepatitis B; yes, no) and gingival bleeding (yes, no, unknown). Fully conditional specification (FCS) methods of multiple imputation was applied to deal with the missing data for confounders [20, 21]. To rule out the potential modification effect of chronic inflammation due to periodontal disease on the association of interest, we conducted subgroup analyses by stratifying whether a woman had frequent gingival bleeding. Kaplan-Meier survival curves and log-rank test were used to assess the bivariate association between halitosis and fecundability within 24 months. All statistical analyses were conducted using SAS version 9.4 (SAS Institute Inc., Cary, NC, USA).

## Results

Among the 3915 women, 3136 (80.1%) and 3369 (86.1%) became pregnant within 12 and 24 months, respectively. The prevalence of self-reported halitosis was 8.7%. The mean age of these women was 28.0 (range 20–49) years. The vast majority was Han ethnicity (98.7%) and had normal weight (73.1%). Those with self-reported halitosis had a lower level of education, and were more likely to smoke, drink, dislike to eat vegetable, and like to eat raw meat. They were also more likely to perceive a higher level of life or work-related stress and financial pressure, have a tense relationship with relatives, friends or colleagues, and a higher prevalence of gingival bleeding (Table 1). They had a slightly longer TTP. 73.1% of women in the halitosis group reported a clinically confirmed pregnancy by 12 months, while 80.8% did so in the non-halitosis group.

**Table 1:** Baseline characteristics of women by halitosis status, *N* = 3915

	Halitosis *N* = 342	Non – halitosis *N* = 3573
**Ethnicity**		
Han	337 (98.5)	3535 (98.9)
Others	5 (1.5)	38 (1.1)
**Age at preconception care (years)**		
< 25	45 (13.2)	409 (11.5)
25–29	181 (52.9)	2083 (58.3)
30–34	106 (31.0)	952 (26.6)
35–39	10 (3.0)	111 (3.1)
≥ 40	0 (0.0)	18 (0.5)
**Educational level (years)**		
≤ 9	13 (3.8)	114 (3.2)
10–12	41 (12.0)	367 (10.3)
13–16	268 (78.4)	2730 (76.4)
≥ 17	20 (5.9)	362 (10.1)
**Occupation**		
Teachers, civil servants and businessmen	161 (47.1)	1791 (50.1)
Farmers, workers and waiter	43 (12.6)	486 (13.6)
Housework or others	138 (40.4)	1296 (36.3)
**BMI at preconception care (kg/m**^**2**^**)**^**a**^		
< 18.5	73 (21.4)	676 (18.9)
18.5–24.9	240 (70.2)	2622 (73.4)
25–29.9	23 (6.7)	220 (6.2)
≥ 30	1 (0.3)	38 (1.1)
Unknown	5 (1.5)	17 (0.5)
**Smoking status**		
Never	329 (96.2)	3542 (99.1)
Yes	13 (3.8)	30 (0.8)
Unknown	0 (0.0)	1 (0.03)
**Alcohol consumption**		
Never	246 (71.9)	2859 (80.0)
Occasionally	91 (26.6)	689 (19.3)
Frequently	5 (1.5)	19 (0.5)
Unknown	0 (0.0)	6 (0.2)
**Dislike to eat vegetables**		
No	319 (93.3)	3462 (96.9)
Yes	23 (6.7)	111 (3.1)
**Like to eat raw meat**		
No	316 (92.4)	3453 (96.6)
Yes	26 (7.6)	120 (3.4)
**Perceived life or work-related stress**		
No	58 (17.0)	917 (25.7)
Yes	284 (83.0)	2656 (74.3)
**Tension with relatives, friends or colleagues**		
No	222 (64.9)	2830 (79.2)
Yes	120 (35.1)	743 (20.8)
**Feeling of financial pressure**		
No	136 (39.8)	1848 (51.7)
Yes	206 (60.2)	1725 (48.3)
**Medical history**^**b**^		
No	272 (79.5)	3093 (86.6)
Yes	70 (20.5)	480 (13.4)
**Gingival bleeding**		
No	105 (40.7)	2239 (62.7)
Yes	178 (52.0)	892 (25.0)
Unknown	59 (17.3)	442 (12.4)
**Time-to-pregnancy [observation period = 12 months; Median (p25, p75)]**		
	6 (3, 12)	6 (2, 12)
**Time-to-pregnancy [observation period = 24 months; Median (p25, p75)]**		
	6 (3, 18)	6 (3, 12)

^a^BMI: body mass index; raw meat: meat is not cooked such as sashimi

^b^Medical history: anemia, hypertension, heart disease, diabetes, thyroid disease, chronic nephritis, tumour, tuberculosis, hepatitis B

**Fig. 2:**
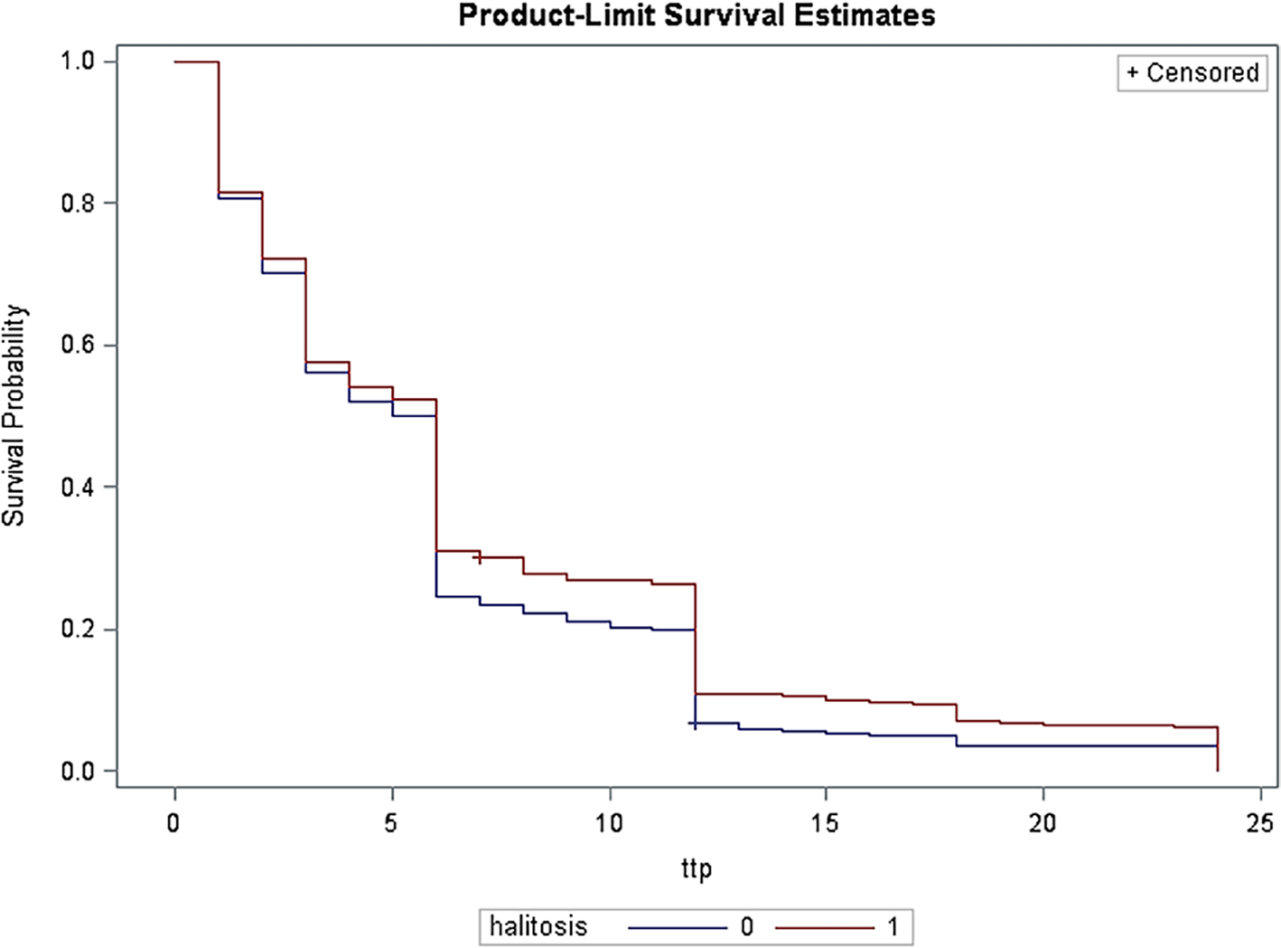
Kaplan-Meier curve for time to pregnancy according to halitosis (yes was recorded as 1, and otherwise 0). Quicker to get pregnant is indicated for women without halitosis (Log-Rank *P* = 0.02)

Table 2 shows that compared with women without halitosis, those with halitosis had reduced fecundability in the observation period of 12 months (adjusted FR 0.82, 95% CI 0.72–0.94) and 24 months (adjusted FR 0.84, 95% CI 0.74–0.96) after controlling for potential confounders. Figure 2 shows that women without halitosis were quicker to become pregnant. Stratified analyses by gingival bleeding further showed that halitosis was associated with decreased fecundability regardless of gingival bleeding (Table 3).

**Table 2:** Multivariate survival analysis of the association between halitosis and time to pregnancy

Exposure Categories	Observation period = 12 months					Observation period = 24 months				
	Pregnancy n (%)	FR	95% CI	^a^aFR	95% CI	Pregnancy n (%)	FR	95% CI	^a^aFR	95% CI
**Halitosis**										
No	2886 (80.8)	Ref	\	Ref	\	3089 (86.5)	Ref	\	Ref	\
Yes	250 (73.1)	0.84	0.74–0.95	0.82	0.72–0.94	280 (81.9)	0.86	0.76–0.97	0.84	0.74–0.96

Abbreviation: *aFR* adjusted fecundability ratio

^a^adjusting for female age at preconception care (years), BMI at preconception care (kg/m^2^), female occupation (teachers, civil servants and businessmen (reference); farmers, workers and waiter; housewife or others), education level (years), smoking status (no, yes), alcohol consumption (no, occasional, frequent, unknown), dislike to eat vegetables (no, yes), like to eat raw meat (no, yes), perceived life or work -related stress (no, yes), tension with relatives, friends or colleagues (no, yes), financial pressure (no, yes), medical history (no, yes), gingival bleeding (no, yes)

**Table 3:** Multivariate survival analysis of the association between halitosis and time to pregnancy stratified by gingival bleeding status

	Observation period = 12 months				Observation period = 24 months			
Exposure Categories	FR	95% CI	^a^aFR	95% CI	FR	95% CI	^a^aFR	95% CI
**Non- Gingival bleeding**								
Non-Halitosis	Ref	\	Ref	\	Ref	\	Ref	\
Halitosis	0.84	0.69–1.03	0.81	0.66–0.995	0.88	0.73–1.06	0.85	0.70–1.03
**Gingival bleeding**								
Non-Halitosis	Ref	\	Ref	\	Ref	\	Ref	\
Halitosis	0.81	0.68–0.98	0.83	0.69–1.00	0.82	0.69–0.97	0.84	0.70–1.00

Abbreviation: *aFR* adjusted fecundability ratio

^a^adjusting for female age at preconception care (years), BMI at preconception care (kg/m^2^), female occupation (teachers, civil servants and businessmen (reference); farmers, workers and waiter; housewife or others), education level (years), smoking status (no, yes), alcohol consumption (no, occasional, frequent, unknown), dislike to eat vegetables (no, yes), like to eat raw meat (no, yes), perceived life or work -related stress (no, yes), tension with relatives, friends or colleagues (no, yes), financial pressure (no, yes), medical history (no, yes)

## Discussion

Our study shows that among Chinese women in Shanghai who planned to be pregnant, 80.1 and 86.1% became pregnant within 12 and 24 months, respectively. We also found that halitosis was negatively associated with female fecundability. Our study was consistent with the other three studies that reported an association of poor oral health and periodontal diseases and prolonged TTP. A case-control study involving 58 non-pregnant women and 70 pregnant women reported that women with good oral hygiene were more likely to be pregnant within 1 year in African American women [10]. A very recent large prospective cohort study in North America indicated a significant association between a self-reported history of periodontitis and decreased fecundability [11]. Hart et al. reported similar results that periodontal disease was associated with prolonged TTP in non-Caucasian (67.1% were Asian and the others were African, Aboriginal, and other ethnicities) women in Australia. But there was no significant association in Caucasian women [9]. The researchers attributed the observed ethnic disparity to the single nucleotide polymorphisms related to periodontal disease [9]. The ethnic disparity in the ability to mount inflammatory/immune responses [9] might be another possible reason. Furthermore, ethnicity might be a proxy for diet, cultural practices, barriers to healthcare access or other structural factors that may affect oral health and fertility.

Microorganisms, especially anaerobic bacteria, are the main cause of bad breath and PD [16–18, 22]. Previous studies showed that oral flora can go through uterine cavity, amnion cavity, placenta, and even fetus, and has been suggested to be an important risk factor for recurrent miscarriage, intrauterine death, preterm birth and neonatal death [23–26]. For example, *Porphyromonas gingivalis*, which plays an important role in the development of halitosis, was found in both periodontal pocket and amniotic fluid of women at high-risk of premature labor [25]. A recent study found that *Porphyromonas gingivalis* infection in female genital tract was associated with recurrent miscarriages [24]. Elevated levels of inflammatory mediators in saliva and some mediators in serum have been found among pre-conception women with periodontal disease [27].

Oral bacteria are also found to be associated with infection at extra-oral locations, such as lung, vasculature and pancreas [28–30]. Bacterial pathogens could activate a cascade of tissue-destructive pathways [31, 32] and elevated systemic levels of cytokines such as tumor necrosis factor alpha, which could lead to chronic systemic inflammation [33]. Such low-grade systemic inflammation is thought to present an endometrial effect similar to endometriosis [10, 34], which could negatively affect fertility and conception. Thus, it is reasonable to hypothesize that increased oral pathogenic bacteria might circulate through bloodstream, enter reproductive organ, and then, result in reduced fecundability [24, 25, 35] .

The present study found a lower prevalence of halitosis (8.7%) in reproductive age women than those in general adult populations in Beijing (27.8%) and Shanghai (33%) [15, 36]. This difference in prevalence may be due to several factors. First, the previous studies measured organoleptic measurements or volatile sulphide compounds, respectively [15, 36], which were likely to be more sensitive than self-report in our study. Thus, some women may have been misclassified in our study. Second, the study populations differed. Our subjects were reproductive-age women, but the subjects in the other studies covered a diverse population [15, 35]. It has been reported that halitosis was more common in those who were older and perceived more stress, but less common in younger females [37, 38]. Iwakura et al. [39] suggested that a person who has halitosis may be accompanied by decreased olfactory function. Thus, underreporting of halitosis in these women is possible. Women may also feel ashamed to report halitosis. However, this was a prospective study. If halitosis is truly associated with infertility, underreporting of halitosis may have drawn our results towards the null to an unknown degree.

Our study was also limited by its inability to explore the underlying causes of halitosis; neither can we prove a cause-and-effect relationship. The self-reported halitosis might be an indicator of bad oral health, or symptoms of other medical conditions and illness, including diabetes, or unhealthy behaviors such as smoking and drinking [4, 15]. However, the significant association remained after we had adjusted for these factors in the multivariable model. We did not have the information on quantity of alcohol consumption, which was a limitation of our study. Thus, the results of our study should be interpreted with caution. Future studies are warranted to explore the underlying mechanisms between the association between halitosis and fecundability.

In addition, 28.5% of subjects were lost to follow-up. We compared women who were lost and who remained in this study. There was no significant difference between these two groups in the prevalence of halitosis (9.2 vs. 8.7, respectively). Thus, the loss-to-follow-up was unlikely to be differential.

## Conclusions

Our large prospective cohort study showed that self-reported halitosis was associated with reduced fecundability in Chinese women who plan to become pregnant. More research is warranted to identify modifiable factors and implement effective prevention strategies to reduce the risk of infertility.

## Supplementary Information

**Additional file 1: Supplementary Figure 1.** Directed acyclic graph illustrated confounders. BMI, body mass index; SES, socioeconomic status.

## Data Availability

All data generated or analyzed during this study are included in this published article.

## Abbreviations

CI: Confidence interval
FR: Fecundability ratio
PD: Periodontal diseases
TTP: Time to pregnancy
