# Supplementary figures and images for: Association between halitosis and female fecundability in China: a prospective cohort study
Source: BMC Pregnancy Childbirth. 2021 Dec 20;21:839. doi: 10.1186/s12884-021-04315-1 (PMC8691089; doi:10.1186/s12884-021-04315-1)

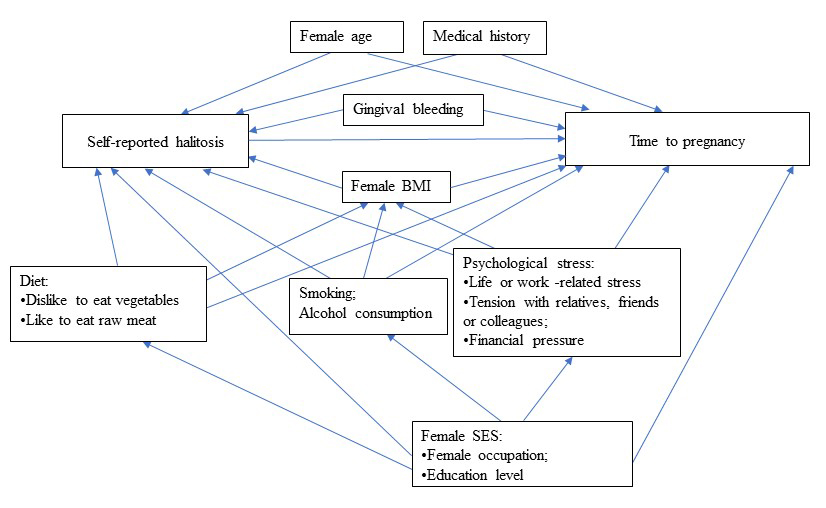

Supplement: Supplementary file 1 — Additional file 1: Supplementary Figure 1. Directed acyclic graph illustrated confounders. BMI, body mass index; SES, socioeconomic status. [file 12884_2021_4315_MOESM1_ESM.tif]
